# A reflection on recent advances in organometallic copper(iii) chemistry

**DOI:** 10.1039/d5sc90259b

**Published:** 2025-12-19

**Authors:** Alicia Casitas, Xavi Ribas

**Affiliations:** a Fachbereich Chemie, Philipps-Universität Marburg 35032 Marburg Germany casitasm@chemie.uni-marburg.de; b mar.quest | Marburg Center for Quantum Materials and Sustainable Technologies 35032 Marburg Germany; c Institut de Química Computacional i Catàlisi (IQCC), Departament de Química, Universitat de Girona, Campus Montilivi Girona 17003 Catalonia Spain xavi.ribas@udg.edu

## Abstract

This commentary provides a broad perspective on recent advances in organometallic copper(iii) chemistry, building on the review we published in 2013 (A. Casitas and X. Ribas, *Chem. Sci.*, 2013, **4**, 2301, https://doi.org/10.1039/C3SC21818J). Parallel with major developments in copper-catalyzed bond-forming reactions, research has increasingly focused on understanding the reactivity of organocopper(iii) complexes. The detailed mechanistic picture tightly depends on the system studied, thus posing challenges to tame the promiscuous behavior of copper catalysis. At the same time, ongoing discussion regarding the formal +3 oxidation state in these compounds, especially those bearing CF_3_ ligands, has provided a deeper understanding of their electronic structures.

## Abstract 2

In celebration of our 15th anniversary and some of our most popular articles, Alicia Casitas and Xavi Ribas reflect on the area of organometallic copper(iii) chemistry, charting developments since their review in *Chemical Science* on this topic: https://doi.org/10.1039/C3SC21818J.

## Graphical abstract

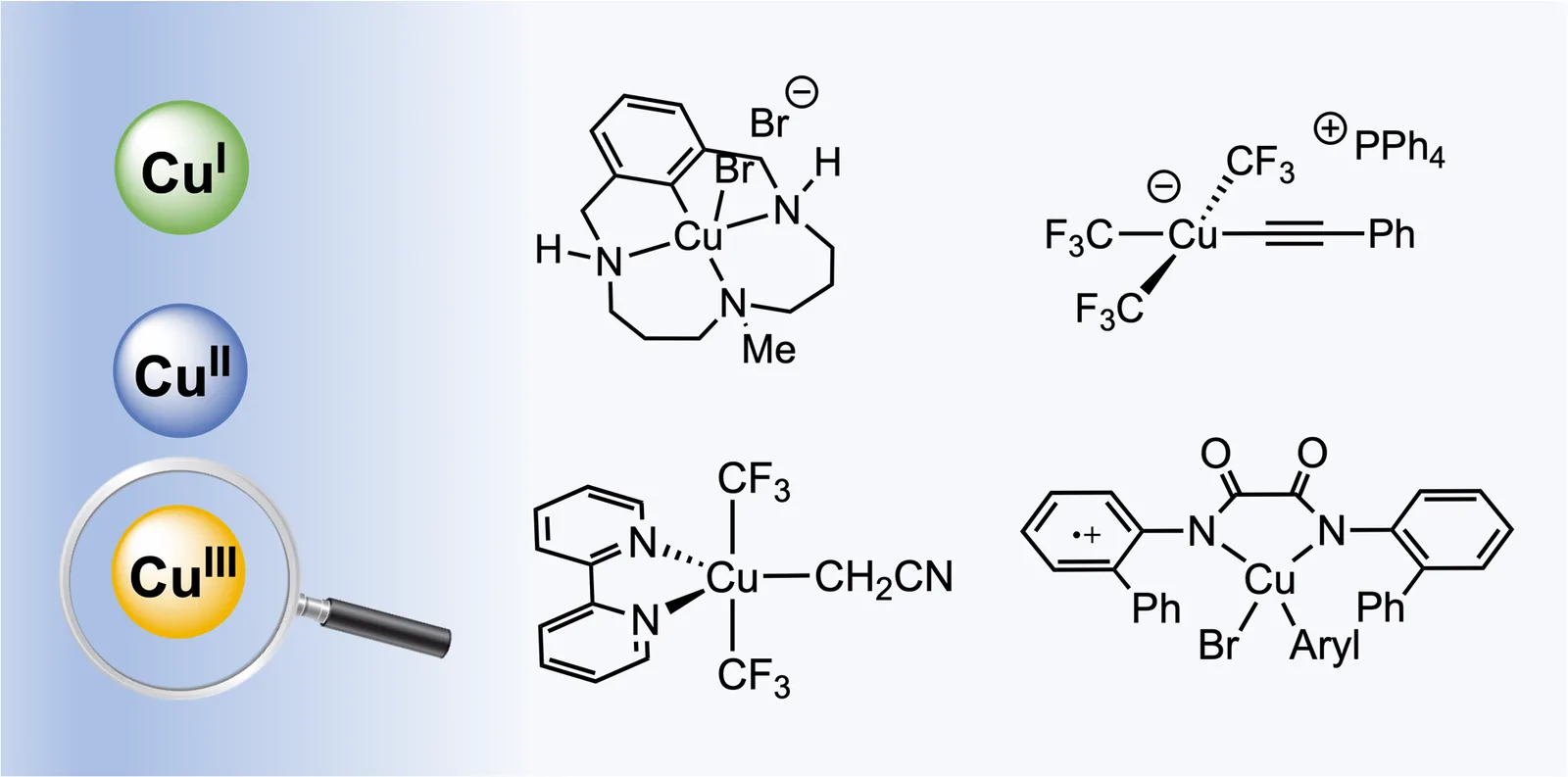

Since our review back in 2013 (https://doi.org/10.1039/C3SC21818J),^1^ the development of organocopper(iii) chemistry has grown considerably.^2^ The synthesis and characterization of new organocopper(iii) complexes during the past decade have been achieved in parallel to major advances in copper catalysis. Ullmann couplings are one of the most relevant reactions catalyzed by copper, together with conjugate additions and click reactions.^3^ Nowadays, a large variety of copper-catalysed C_sp^2^_–C_sp^2^_ and C_sp^2^_–heteroatom bond-forming reactions between several nucleophiles and aryl or vinyl halides are included within the name of Ullmann(-type) couplings. The most invoked mechanistic proposal for Ullmann reactions involves the formation of organocopper(iii) intermediates *via* an oxidative addition process from copper(i) to aryl electrophiles, followed by a fast reductive elimination step that releases the bond-forming product.^4^ Initially, researchers aimed at isolating such highly reactive organocopper(iii) complexes to study their intrinsic reactivity and support the proposed two-electron redox Cu(i)/Cu(iii) catalytic cycle for Ullmann couplings. Early work in isolating copper(iii) complexes focused on the design of nitrogen- and oxygen-based ligands that imposed the square-planar geometry at Cu(iii) to enhance their stability. This approach enabled the isolation of well-defined arylcopper(iii), most in macrocyclic ligands, which were investigated in C_sp^2^_–C_sp_ and C_sp^2^_–heteroatom (C–N, C–O, C–S, C–Se, C–P, C–halogen) reductive elimination reactions with several nucleophiles (amides, phenols, thiols, selenols, phosphines, halides).^5^ Not only was reductive elimination proven at these arylcopper(iii) complexes, but also intramolecular oxidative addition of copper(i) into model macrocyclic aryl halides to form arylcopper(iii).^5*b*^

However, the debate on the reaction mechanism of Ullmann-type couplings is still open. One could argue that the ligands used to stabilize copper(iii) complexes differ markedly from those typically employed in Ullmann-type reactions, which commonly involve diamines, 1,3-amino acids, 1,3-diketones, and oxalamides, among others.^6^ Since the reactivity of the copper center is highly sensitive to the ligand environment, the large variety of ligands used in catalytic conditions – together with the promiscuity of copper's behaviour – suggest that multiple mechanistic scenarios might be accessible depending on the specific reaction conditions. This complexity makes it challenging to depict one general catalytic cycle for Ullmann-type reactions. In this regard, Hartwig and co-workers performed mechanistic studies on the C–O cross-coupling reaction between aryl halides and phenols catalysed by copper(ii) complexes bearing dianionic oxalamide ligands (Fig. 1a),^7^ which are one of the most active ligands for Ullmann-type couplings as shown by Dawei Ma.^8^ The oxalamide ligands are redox non-innocent, and the oxidative addition at copper(ii) affords a Cu(iii) radical cation at the ligand. This example shows that Ullmann couplings can be accommodated into alternative Cu(ii)/Cu(iii) catalytic cycles. In another example, Peters' group showed that photoactive copper(ii) complexes catalyse C–N cross-coupling reactions between nitrogen nucleophiles and aryl and alkyl halides *via* a Cu(i)/Cu(ii) catalytic cycle, which is initiated by the activation of the electrophile by a single-electron transfer (SET) process (Fig. 1b).^9^

**Fig. 1:**
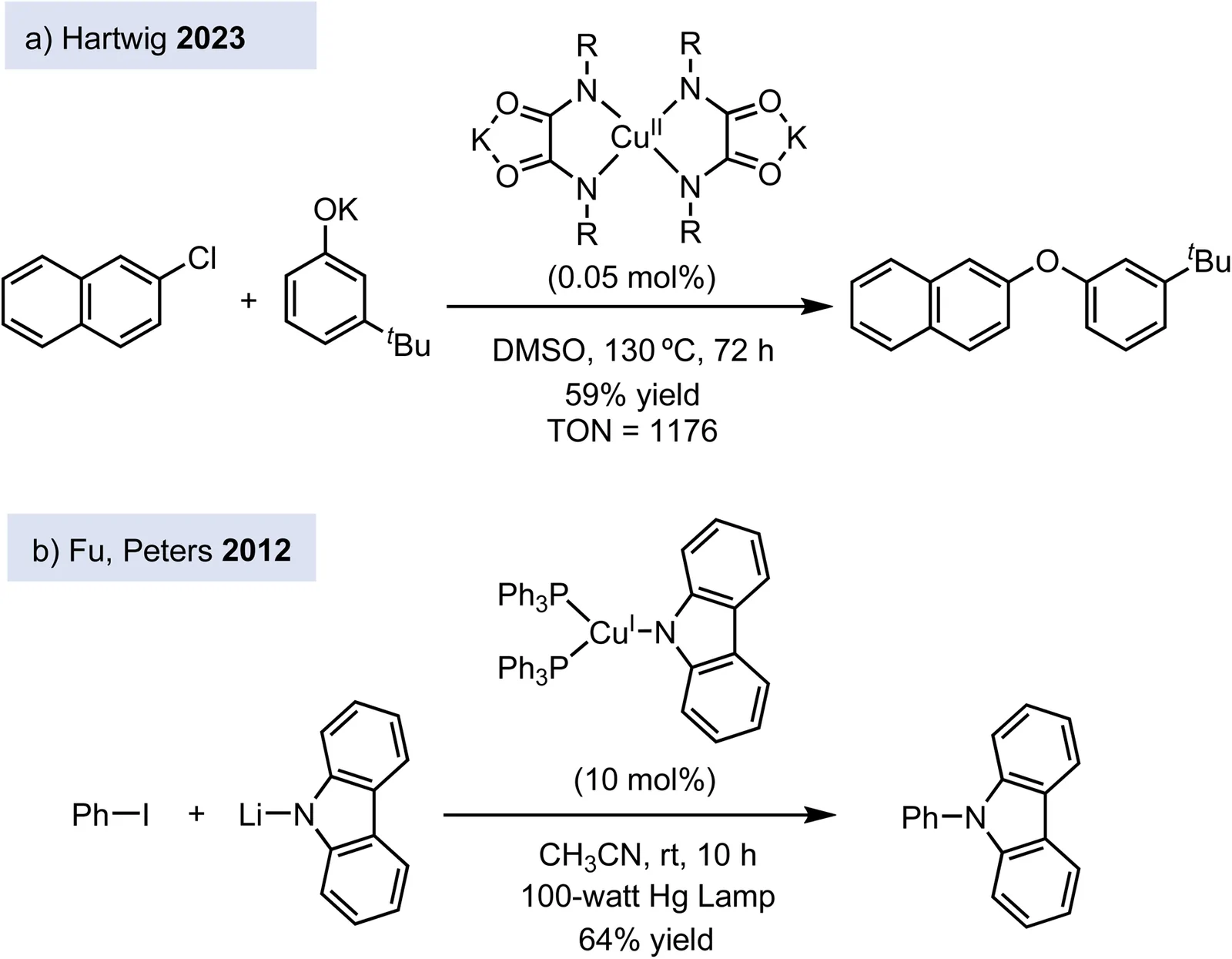
Recent copper-catalysed C–N and C–O cross-coupling reactions by non-canonical mechanisms.

Since 2013, the introduction of perfluoroalkyl ligands has enabled the stabilization and synthesis of a broad family of organocopper(iii) complexes. Although the formal oxidation state assignment in these compounds has been debated (see the end of this commentary), they have been used as mechanistic platforms to gain insight into several fundamental steps in copper chemistry. In particular, several anionic tris(trifluoromethyl) copper(iii) complexes of general formula [Cu(CF_3_)_3_R][Y] (Y^+^ = PPh_4_ or *n*-Bu_4_N), bearing a fourth organic ligand R, such as alkyl group or electron-deficient arene, have been synthesized.^10^ Progress has also been made towards the preparation of neutral trifluoromethylated copper(iii) complexes of type [(L)Cu(CF_3_)_3_], bearing dative N-based ligands such as pyridine (py), 2,2′-bipyridine (bpy), phenanthroline (phen),^11^ and even the weakly coordinating acetonitrile.^12^ In addition, [(L)Cu(CF_3_)_3_] are good surrogates to prepare related copper(iii) complexes bearing one different carbon-based ligand such as [(bpy)Cu(CF_3_)_2_R] (R = Me, C_6_F_5_)^13^ and [(tpy)Cu(CF_3_)_2_R] (R = CH_2_CO_2_^*t*^Bu).^14^ In terms of reactivity, [(L)Cu(CF_3_)_3_] (L = py, bpy, phen) complexes engage in stoichiometric trifluoromethylation reactions of boronic acids (ArB(OH)_2_) to afford trifluoromethyl arenes in the presence of potassium fluoride (KF) (Fig. 2a).^11*b*^

**Fig. 2:**
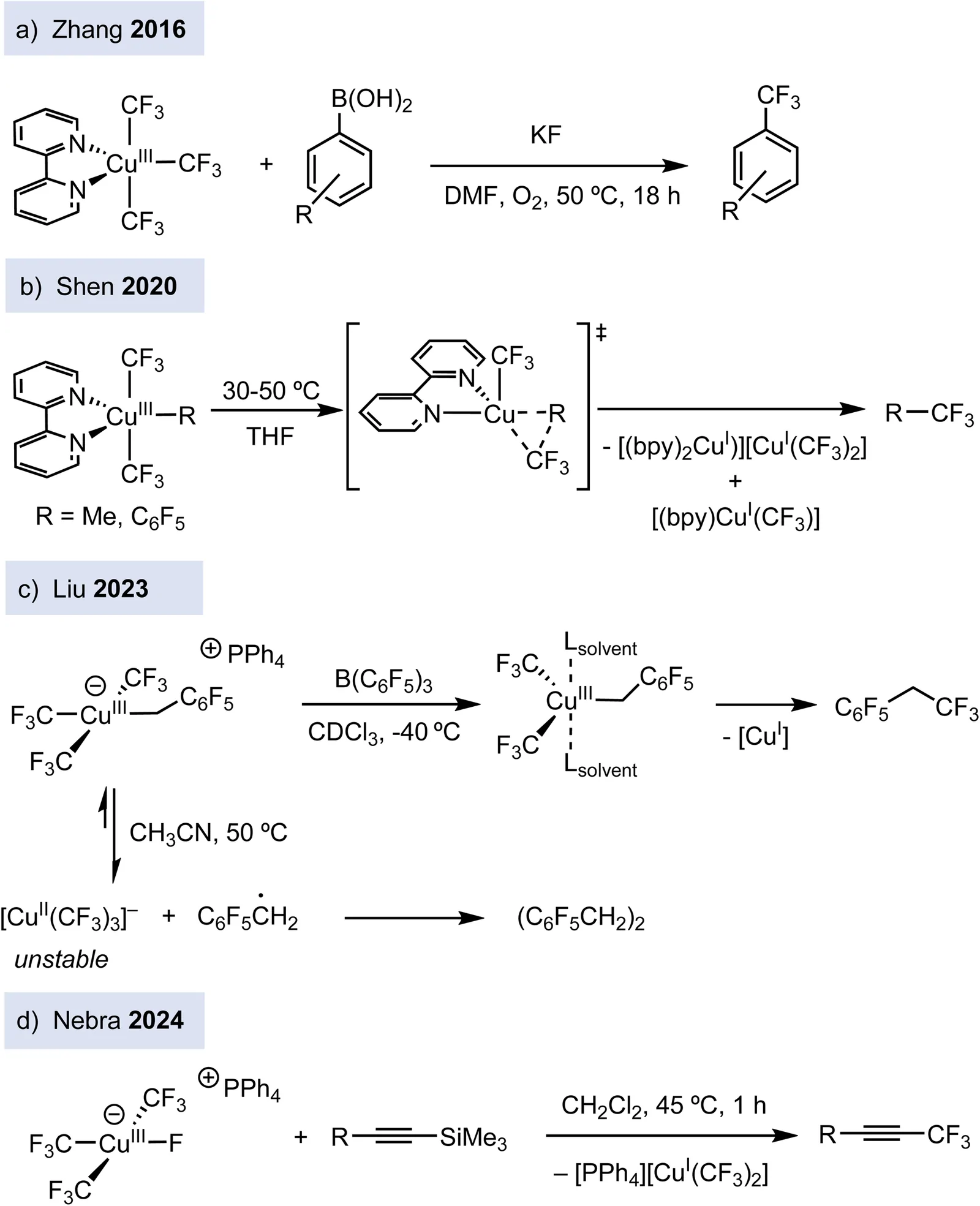
Varied reactivity of trifluoromethylated copper(iii) complexes.

These trifluoromethylated Cu(iii) derivatives have provided mechanistic comprehension on the bond-forming step of trifluoromethylation reactions. For instance, five-coordinated [(bpy)Cu(CF_3_)_2_(R)] (R = CH_3_, C_6_F_5_] and anionic [Cu(CF_3_)_3_R][Y] complexes undergo C_sp^2^_–CF_3_ or C_sp^3^_–CF_3_ bond-forming reactions *via* concerted reductive elimination (Fig. 2b).^10,13^ In contrast, it has been shown that in other complexes, such as [Cu(CF_3_)_3_(CH_2_C_6_F_5_)], homolysis of the Cu–C bond to release ·CH_2_C_6_F_5_ benzylic radicals is the major decomposition pathway (Fig. 2c).^15^ Thus, subtle structural changes in trifluoromethylated copper(iii) translate into different reactivity, highlighting the rich reactivity modes of copper(iii). Further studies showed that the complexes [(L)Cu(CF_3_)_3_] (L = py, phen) react with terminal alkynes to give either *syn*-fluoro trifluoromethylated alkenes or trifluoromethylated alkynes depending on the reactions conditions.^11*c*,16^ [(Py)Cu(CF_3_)_3_] is a good precursor to synthesize the anionic organocopper(iii) fluoride complex [Cu(CF_3_)_3_F][PPh_4_].^17^ The latter not only reacts with silyl-capped alkynes to trigger C_sp_–CF_3_ bond-formation (Fig. 2d), but it can also be derivatized to ten different trifluoromethylated copper(iii) complexes. Finally, it has been shown that the [(bpy)Cu(CF_3_)_3_] complex enables the C_sp^3^_–H trifluoromethylation of alkanes.^18^ After light irradiation, [(bpy)Cu(CF_3_)_3_] undergoes CF_3_ radical-mediated HAT reaction into C_sp^3^_–H bonds, followed by radical-polar crossover and ionic coupling.

During the past decade, we have witnessed the development of recent methodologies in copper-catalyzed reactions beyond Ullmann-type couplings, such as benzylic and allylic C–H functionalizations,^19^ difunctionalization of alkenes,^20^ and cross-coupling reactions with C_sp^3^_–X electrophiles, such as alkyl halides.^21^ Furthermore, dual visible-light photoredox and copper catalysis towards a variety of bond-forming reactions have been developed, including also trifluoromethylation reactions.^22^ These methodologies have leveraged the use of radical precursors (such as aryl halides, carboxylic acids, redox active esters) to form carbon-centered radicals by means of a photoredox catalyst, or by hydrogen atom abstraction reagents that enable the direct activation of C_sp^3^_–H bonds. In many of these protocols, the bond-forming event is usually proposed to occur through the reaction of a copper(ii) intermediate – bearing a specific functional group – with a carbon-centered radical, leading to the release of the final coupling product. One commonly postulated pathway for this key transformation involves an inner-sphere oxidative substitution of the radical onto the Cu(ii) complex, which forms a formal Cu(iii) intermediate that subsequently undergoes reductive elimination. Alternative mechanisms have also been proposed such as outer sphere direct atom transfer of the functional group on the copper(ii) to the radical, or direct oxidation of the alkyl radical by Cu(ii) species, forming Cu(i) and a carbocation that is attacked by a nucleophile.^23^ Additionally, the development of asymmetric C_sp^3^_–H functionalizations and C_sp^3^_–C cross-coupling of racemic alkyl electrophiles has also been accomplished, provided a specific chiral ligand is coordinated to the copper.^24^ Here, the stereoselectivity is proposed to arise from the reaction of the planar radical with the chiral copper(ii)–nucleophile intermediate.^19*a*^ However, experimental data that support the feasibility of synthesizing well-defined organocopper(iii) complexes from the reaction of carbon-centered radicals with copper(ii) complexes are narrow. One specific example is the *trans* to *cis* isomerization process involving trifluoromethylated copper(iii) complexes.^14^

Exploiting the strategy of stabilizing copper(iii) complexes bearing trifluoromethyl groups, recent efforts have focused on studying the reactivity of copper(i) precursors with organic halides. In contrast to earlier oxidative addition studies on model macrocyclic aryl halide ligands by Ribas, Stahl, and Wang – which were limited to intramolecular examples – the use of trifluoromethylated copper(i) complexes enables the investigation of intermolecular oxidative addition processes of a wider range of organic halide electrophiles. Indeed, electron-deficient alkyl halides are reactive electrophiles that facilitate the oxidative addition step to Cu(i), leading to the kinetically stable and isolable organocopper(iii) complexes, as reported by Hartwig and Shen's groups. Anionic and neutral copper(i) complexes, [Cu(CF_3_)_2_][PPh_4_] and [(bpy)Cu(CF_3_)], undergo oxidative addition with α-haloacetonitriles (XCH_2_CN, X = Cl, Br, I) to form the corresponding alkylcopper(iii) complexes (Fig. 3a).^25^ Experimental and theoretical studies support that complex [Cu(CF_3_)_2_][PPh_4_] reacts with α-bromoacetonitrile following an S_N_2 mechanism (with minor contribution of halogen atom transfer (XAT)), whereas [(bpy)Cu(CF_3_)] reacts mainly through the XAT pathway. This study suggests that a free radical could be involved in the oxidative addition of an alkyl halide to Cu(i) to form a Cu(iii) intermediate through the XAT pathway.

**Fig. 3:**
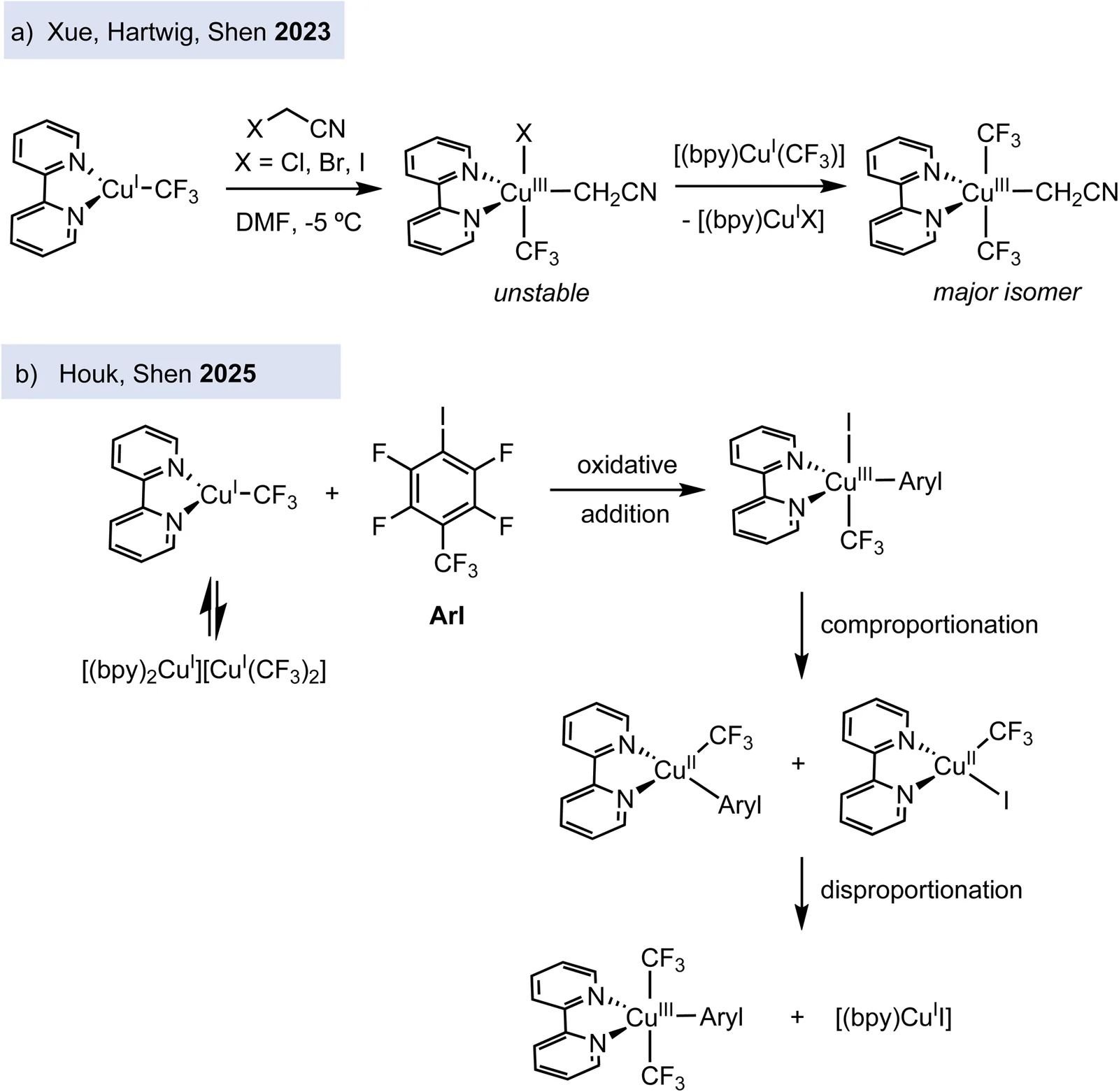
Reactivity of copper(i) with electron-deficient alkyl and aryl halides.

Very recently, the reaction of [(bpy)Cu(CF_3_)] with an electron-deficient aryl iodide, *i.e.* 1,2,4,5-tetrafluoro-3-iodo-6-(trifluoromethyl)benzene (*p*CF_3_C_6_F_4_I), has been investigated at low temperature by Houk and Shen (Fig. 3b).^26^ This work shows that oxidative addition of the aryl iodide to Cu(i) affords the organocopper(iii) [(bpy)Cu(Ar)(I)(CF_3_)], which reacts fast with the initial [(bpy)Cu(CF_3_)] in a comproportionation reaction to give two Cu(ii) intermediates, [(bpy)Cu(CF_3_)(I)] and [(bpy)Cu(CF_3_)(Ar)] (both detected by EPR spectroscopy). The transient Cu(ii) complexes disproportionate to form a final Cu(iii) complex, [(bpy)Cu(CF_3_)_2_(Ar)], and the Cu(i) complex [(bpy)Cu(i)], proceeding most likely *via* trifluoromethyl radical transfer through either inner-sphere radical rebinding process. Although the mechanistic investigations were performed with a specific electron-deficient iodoarene, this work reveals that formal oxidative addition organocopper(iii) products might be formed through more complex redox scenarios, involving several oxidation states such as +1, +2 and +3.

However, the actual definition of the oxidation state at formal organocopper(iii) complexes is an ongoing matter of debate since the seminal work of Snyder in 1995, who questioned the oxidation state +3 of the anionic complex [Cu(CF_3_)_4_]^−^.^27^ Snyder suggested that [Cu(CF_3_)_4_]^−^ is better described as d^10^ Cu(i) instead of a d^8^ Cu(iii) center, based on a computational study that gave a partial charge on Cu of 0.71 and d-orbital populations of 9.7 or 9.4 (close to 10 count for Cu(i)). Thus, the Cu(i) would be coordinated to three CF_3_^−^ ligands and one CF_3_^+^ ligand. Owing to resonance, this would best be described as delocalized CF_3_^+^ over the molecule, thus a Cu(i) surrounded by a (CF_3_)_4_^2−^ coordination sphere. This picture is referred to as “inverted ligand field”, in which the lowest unoccupied molecular orbital (LUMO) exhibits dominantly ligand orbital admixture, contrary to the classical ligand field in which the LUMO frontier orbitals are dominated by metal d-orbital admixture.^28^ This description has been supported computationally and experimentally by the groups of Lancaster,^29^ Klein,^30^ Overgaard^31^ and Mankad.^32^ The use of Cu-K-edge synchrotron X-ray Absorption Spectroscopy (XAS) as a diagnostic tool for the analysis of these formally organocopper(iii) complexes is controversial. While the weak quadrupole-allowed pre-edge feature occurring at 8981 ± 0.5 eV was assigned to a 1s → 3d transition at d^8^ Cu^III^ complexes, later it was shown that some Cu^II^ complexes can also exhibit the 1s → 3d transition in the same range, hampering the assignment of the oxidation state of the copper by this spectroscopic technique. Lancaster and co-workers have proposed that Cu L_2,3_-edge XAS, together with experimentally calibrated electronic structure calculations, is an appropriate tool to investigate formally organocopper(iii) complexes, which in most cases exhibit ligand field inversion.^29*b*^ We envision that this will continue as an open discussion until a satisfactory explanation is provided to address the conflicting arguments.^33^

Regardless of the ongoing debate about the oxidation state +3 on the copper, the research outlined here on organometallic copper complexes with unusual electronic structures goes beyond mere chemical curiosities. Organocopper(iii) complexes are highly reactive, making it challenging to study them through *in situ* spectroscopic methods to unravel their role as catalytic intermediates under synthetically relevant reaction conditions. Thus, performing reactivity studies using designed copper(iii) complexes has been instrumental in elucidating their role in bond-forming and bond-cleavage processes. These mechanistic insights have, in turn, guided the development of novel copper-catalyzed reactions towards organic synthesis. Despite these advances, the field still offers considerable opportunities for progress and innovation. Future directions include: the design and synthesis of new stabilizing ligands for copper(iii) complexes beyond macrocyclic N-based ligands and perfluoroalkylated groups, along with the elucidation of their electronic structures through spectroscopic methods; the study of oxidative addition processes at new copper(i) systems to form copper(iii) across a broader range of electrophiles; the exploration of copper(ii) as a radical trap to synthesize and isolate copper(iii); and the examination of the photochemical properties of organocopper(iii) complexes. Equally relevant are comprehensive mechanistic and kinetic investigations to support proposed catalytic cycles for newly developed copper-catalyzed reactions, and to underpin the bottom-up discovery of novel transformations. We anticipate that this area of research will remain vibrant for years to come.

## Author contributions

A. C. and X. R. wrote together the manuscript.

## Conflicts of interest

There are no conflicts to declare.

## Data Availability

No primary research results, software or code have been used in this Commentary.
